# Coactivation of MEP*-*biosynthetic genes and accumulation of abietane diterpenes in *Salvia sclarea* by heterologous expression of WRKY and MYC2 transcription factors

**DOI:** 10.1038/s41598-018-29389-4

**Published:** 2018-07-20

**Authors:** Mariaevelina Alfieri, Maria Carmela Vaccaro, Elisa Cappetta, Alfredo Ambrosone, Nunziatina De Tommasi, Antonietta Leone

**Affiliations:** 1 Department of Pharmacy, University of Salerno, Via Giovanni Paolo II 134D, 80084 Fisciano, Italy; 2 Present Address: Department of Agricultural Sciences, University of Naples Federico II, via Università 100, 80055 Portici (Naples), Italy

## Abstract

Plant abietane diterpenoids (*e*.*g*. aethiopinone, 1- oxoaethiopinone, salvipisone and ferruginol), synthesized in the roots of several *Salvia* spp, have antibacterial, antifungal, sedative and anti-proliferative properties. Recently we have reported that content of these compounds in *S*. *sclarea* hairy roots is strongly depending on transcriptional regulation of genes belonging to the plastidial MEP-dependent terpenoid pathway, from which they mostly derive. To boost the synthesis of this interesting class of compounds, heterologous *At*WRKY18, *At*WRKY40, and *At*MYC2 TFs were overexpressed in *S*. *sclarea* hairy roots and proved to regulate in a coordinated manner the expression of several genes encoding enzymes of the MEP-dependent pathway, especially DXS, DXR, GGPPS and CPPS. The content of total abietane diterpenes was enhanced in all overexpressing lines, although in a variable manner due to a negative pleiotropic effect on HR growth. Interestingly, in the best performing HR lines overexpressing the *At*WRKY40 TF induced a significant 4-fold increase in the final yield of aethiopinone, for which we have reported an interesting anti-proliferative activity against resistant melanoma cells. The present results are also informative and instrumental to enhance the synthesis of abietane diterpenes derived from the plastidial MEP-derived terpenoid pathway in other *Salvia* species.

## Introduction

*Salvia sclarea* is a cash-crop primarily cultivated for the extraction of scareol, a byciclic diterpenes, used as a fragrance in cosmetics and perfumes and as flavoring in food. Additional bioactive abietanes synthesized in the roots of this species and other *Salvia* species have shown a broad array of biological effects, such as antibacterial, antioxidant, anti-inflammatory, antifungal^1^.

Abietane diterpenes have been also proved to have an anti-proliferative activity against several tumour cell lines: salvipisone and aethiopinone from *S*. *sclarea* induce apoptosis in a time- and concentration-dependent manner in leukemia cells^2^. We have recently shown that aethiopinone, purified from *S*. *sclarea* hairy roots^3^, has an anti-proliferative and apoptotic activity against different solid tumour cell lines, especially against melanoma A375 cells^3^, the most aggressive form of skin cancer for which advanced stages are inevitably resistant to conventional therapeutic agents^4^.

Despite their promising biological activities, the potential translation of these molecules into novel pharmacological anti-tumour drugs is hampered by the low accumulation rate in natural producing plants, a common drawback of most of the plant-derived secondary metabolites, which even prevents more accurate pre-clinical assays and clinical tests. Additionally, in many cases synthetic procedures do not ensure sufficient amount, stability and purity of these compounds.

**Figure 1:**
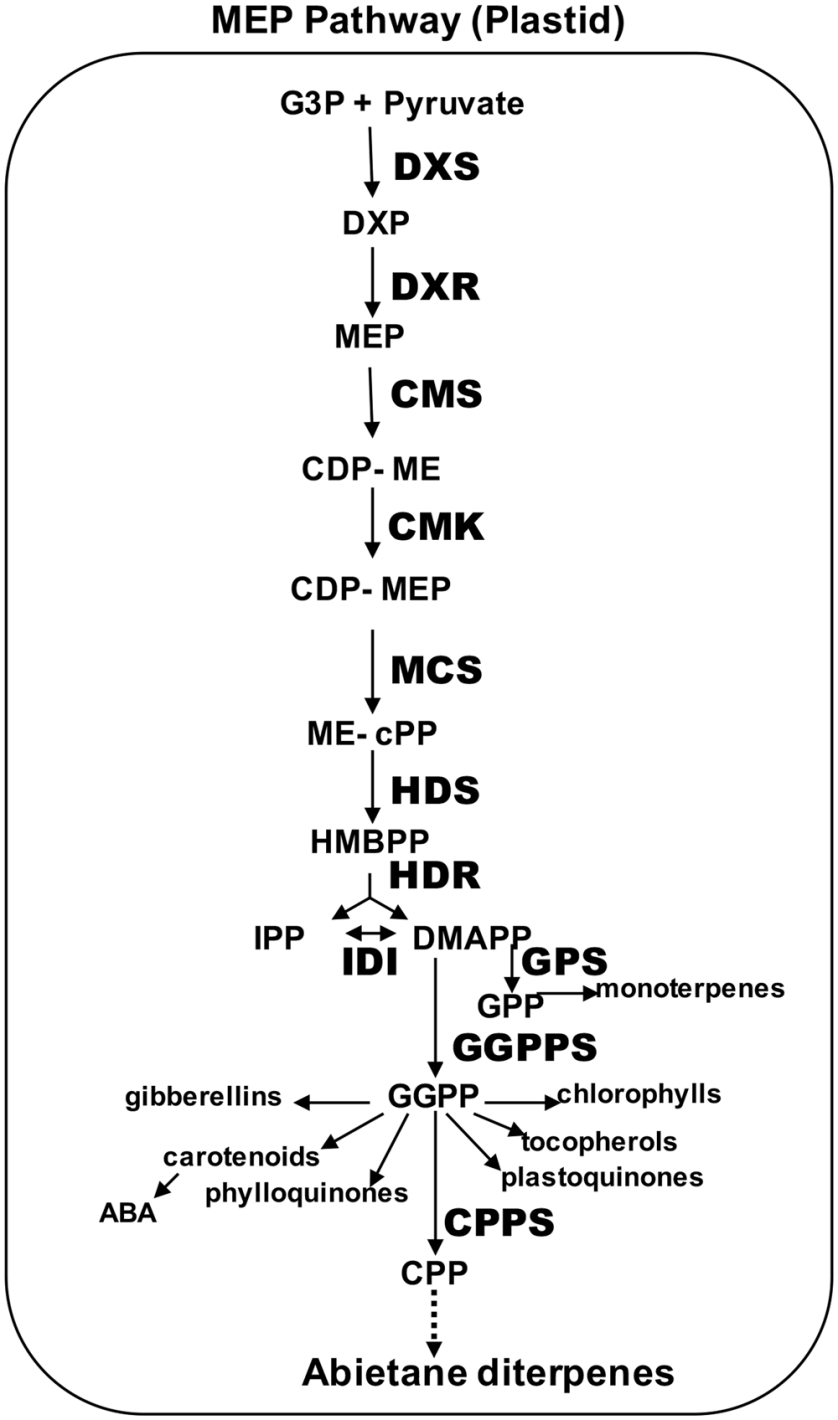
Schematic representation of the plastidial MEP-derived terpene pathway. The main enzymatic steps (bold capital letters), intermediates and final products (regular letters) are indicated. Abbreviations: DXS, deoxyxylulose 5-phosphate synthase; DXR, deoxyxylulose 5-phosphate reductoisomerase; CMS, 4-diphosphocytidyl-methylerythritol synthase; CMK, 4-diphosphocytidyl-methylerythritol kinase; MCS, methylerythritol 2,4-cyclodiphosphate synthase; HDS, hydroxymethylbutenyl 4-diphosphate synthase; HDR, hydroxymethylbutenyl 4 diphosphate reductase; GPPS, GPP synthase; GGPPS, GGPP synthase; IDI, isopentenyl diphosphate isomerase; CPPS, CPP synthase; IPP, isopentenyl pyrophosphate; DMAPP, dimethylallyl pyrophosphate; GPP, geranyl pyrophosphate; GGPP, geranylgeranyl pyrophosphate; CPP, copalyl diphoshate.

Recently, we have used hairy root (HR) technologies in combination with metabolic engineering strategies, and provided one of the first evidences that the production of tryciclic abietane diterpenes can be enhanced by 3–4 times by overexpressing in *S*. *sclarea* hairy roots the heterologous genes *DXS* or *DXR* from *Arabidopsis thaliana*^3^. We have also demonstrated that the expression of the genes encoding these two enzymes, acting up-stream of geranyl-geranyl disphosphate (GGPP), the common precursor of most of the MEP (2-*C*-methyl-D-erythritol 4-phosphate) pathway-derived terpenes (Fig. 1), are transcriptionally regulated by methyl jasmonate (MJ), along with other genes acting upstream and downstream of GGPP^5^. Significant correlations were found between the content of total abietane diterpenes and the level of expression of the *DXS*, *DXR* and *GGPPS* genes as well as of the *CPPS* gene (copalyl disphosphate synthase), the first enzyme involved in the synthesis of copalyl disphospate, precursor of several plant abietane diterpenes^6^. Altogether this set of data indicates clearly the existence of a MJ-dependent gene regulation of the MEP-derived terpene biosynthetic route.

In this work, we aimed at enhancing the content of abietane diterpenes production in *S*. *sclarea* HRs by overexpressing transcription factors (TFs), whose expression is known to be MJ-inducible, and that might be potentially involved in the regulation of the MEP-pathway genes. Recent findings have elucidated the role of some TFs in the biosynthesis of plant secondary metabolites, including terpenes, as reported for the sesquiterpene artemisinin in *Artemisia annua* (*AaWRKY1*, *AaERF1*, *AaERF2*, *AaORA1 and AabZIP1*)^7–10^. Similar evidences have been also obtained for other TFs, such as *Os*TGAP1, which controls the synthesis of phytocassanes, a class of diterpene phytoalexins in rice^11^*; Ga*WRKY1, regulating gossypol biosynthesis in cotton^12^ and *Ta*WRKY1 controlling the regulation of 10-deacetylbaccatin III-10-O-acetyl transferase involved in taxol production in *Taxus chinensis*^13^.

Interestingly, several TFs regulating secondary metabolism are MJ-responsive^14^, among which, MYC2 is one of the best characterised. It belongs to the basic helix-loop-helix (bHLH), which positively or negatively regulates secondary metabolism during JA signalling in a species-specific manner. The basic region of the MYC2 protein, containing 15–20 mostly basic amino acids, is involved in binding the conserved G-box (5′-*CACGTG*-3′) found in the promoters of MJ-responsive genes, by forming homo- and/or heterodimers with other members of the MYC family, such as MYC3 and MYC4^15^.

Another emerging class of TFs involved in the regulation of plant secondary metabolites is the large WRKY family, a plant specific class of TFs, firstly discovered for their role in abiotic and biotic plant response^16^. Accumulating evidence suggests that certain WRKYs regulate the production of valuable natural products by modulating transcriptionally the biosynthetic genes involved in the synthesis of phenylpropanoids, alkaloids and terpenes^17^. Although some WRKY proteins may contain additional domains, they all have a common highly conserved DNA-binding domain (DBD)^18^ of ∼60 amino acids characterised by the conserved heptad WRKYGQK amino acid motif at their N-terminus, which may vary in number, and a zinc ion chelating finger structure at their C-terminus. WRKY TFs bind their cognate (*T*)*TGAC*)*C/T*) W-box cis-elements in the promoter region of target genes^19^.

Owing to the limited available information on *S*. *sclarea* genome and transcriptome, we used *Arabidopsis thaliana* as a genetic source of potential TFs involved in MJ-dependent regulation of the biosynthetic genes of the MEP-pathway, relying on the fact that the structural organization of TFs is largely conserved in the plant kingdom.

The WRKY TF family has been well characterized for their involvement in SA signalling and plant defense in Arabidopsis, but their role remains less clear for jasmonate signalling, especially for their contribution in the regulation of secondary metabolism^20^. Among several members belonging to the large WRKY TF families we focused our attention on the *At*WRKY18, *AtWRKY40* TFs as either publically available microarray datasets and published resulted have demonstrated that they are involved in the methyl jasmonate signalling pathway^21^, where they have been suggested to operate also with overlapping roles^22^. In addition, a recent genome wide binding analysis of WRKY18 and 40 detected WRKY binding sites in several genes encoding functions related to MJ signalling^22–27^.

More robust evidences are available for the involvement of *At*MYC2 as the best characterized and most multifunctional TFs, acting as a regulatory hub within the JA signalling pathway^15^. Furthermore, a genome-wide search has revealed that 25% of early JA-responsive genes (i.*e*. genes responding to JA treatment within 30 min of JA application) contain the G-box *cis*-acting sequence, providing additional support for the potential importance of this sequence in MYC2-regulated expression of JA-responsive genes^28^.

Herein, we confirmed that in *A*. *thaliana* the expression of these three TFs is tightly controlled by MJ elicitation and precedes the activation of the biosynthetic genes of the MEP-pathway from which abietane diterpenes derive. In addition, by a preliminary promoter scanning analysis, several W- and G-cis-elements were identified in the promoters of the biosynthetic genes encoding enzymes of the MEP-pathway. Thereafter, we proved that the ectopic expression of *At*WRKY18, *At*WRKY40 and *At*MYC2 activates in a coordinated manner the transcription of several genes of the MEP-pathway in *S*. *sclarea* HRs, which, in turn, accumulated significantly higher contents of abietane compounds.

## Results

### *A. thaliana* MEP-pathway genes are MJ inducible and contain putative binding sites of known transcription factors in their promoters

**Figure 2:**
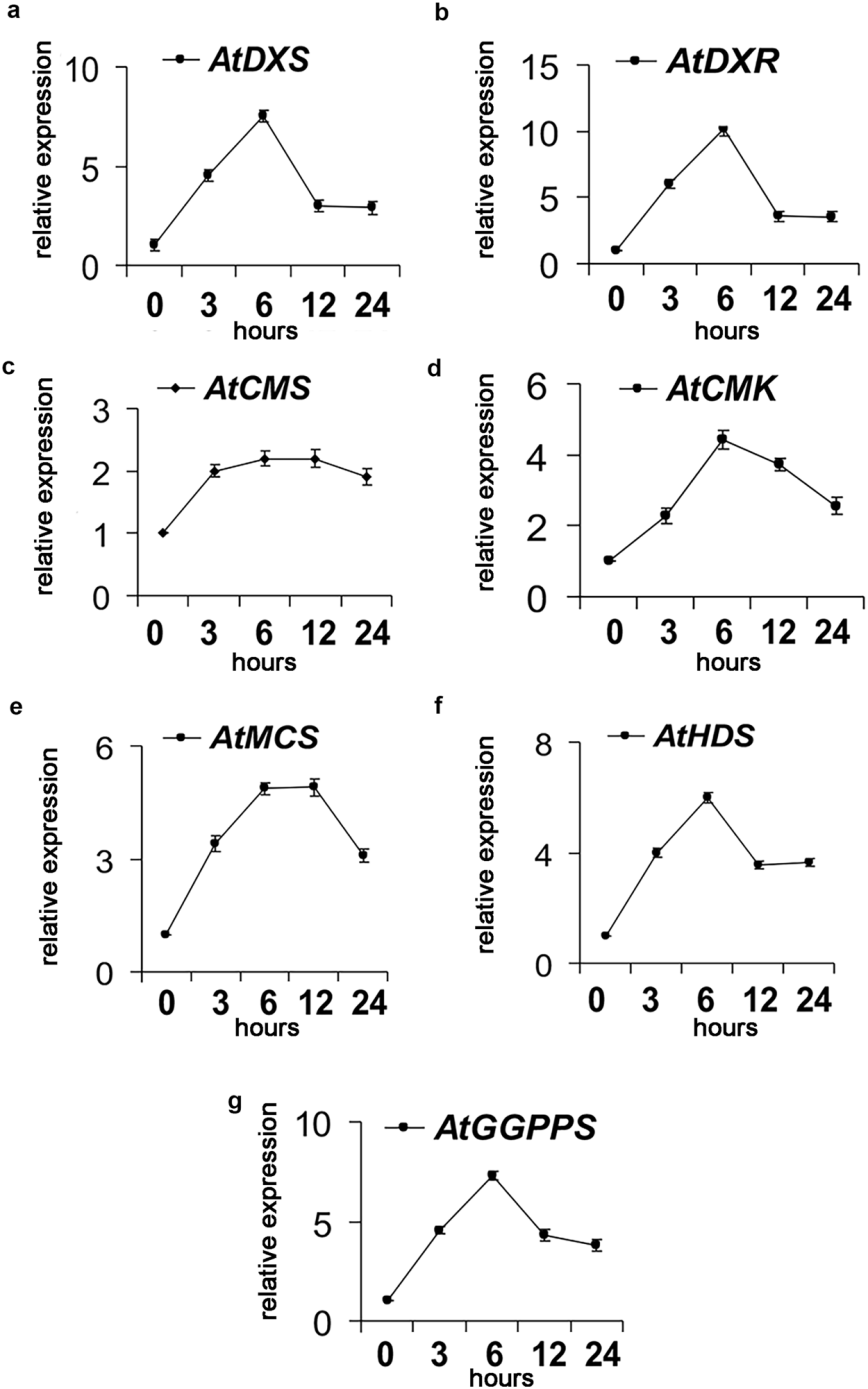
Time-course expression analyses of *AtDXS*, *AtDXR*, *AtCMK*, *AtMCT*, *AtHDS*, *AtGGPPS* genes in 14-day old *A*. *thaliana* plantlets treated with 150 M MJ for 24 h, measured by qRT-PCR and expressed as fold-changes compared to the expression level of untreated control seedlings. 18S rRNA was used to normalize the expression of TFs.

A time-course experiment (24 h) in MJ-treated *Arabidopsis* plantlets revealed that all the biosynthetic genes of the terpene MEP-derived pathway, leading to the common precursor GGPP, are transcriptionally activated by this elicitor (Fig. 2a–f), namely *deoxyxylulose 5-phosphate synthase* (*DXS*), *deoxyxylulose 5-phosphate reductoisomerase* (*DXR*), *4-diphosphocytidyl-methylerythritol kinase* (*CMK)*, *4-diphosphocytidyl-methylerythritol synthase* (*MCS)*, *hydroxymethylbutenyl 4-diphosphate synthase* (*HDS)*, *geranyl geranyl pyrophosphate synthase* (*GGPPS)*. All transcripts were transiently up-regulated in *A*. *thaliana* seedlings treated with 150 μM MJ compared to control untreated seedlings. Overall, transcriptional activation was robust and initiated within 3 h post-treatment, reaching the peak after 6 h. Upon MJ elicitation, *AtDXS*, *AtDXS* and *AtGGPPS* were the most induced transcripts (fold change > 8), although at lower levels as we have found in MJ-treated *S*. *sclarea* HRs^5^ (Table S1).

**Figure 3:**
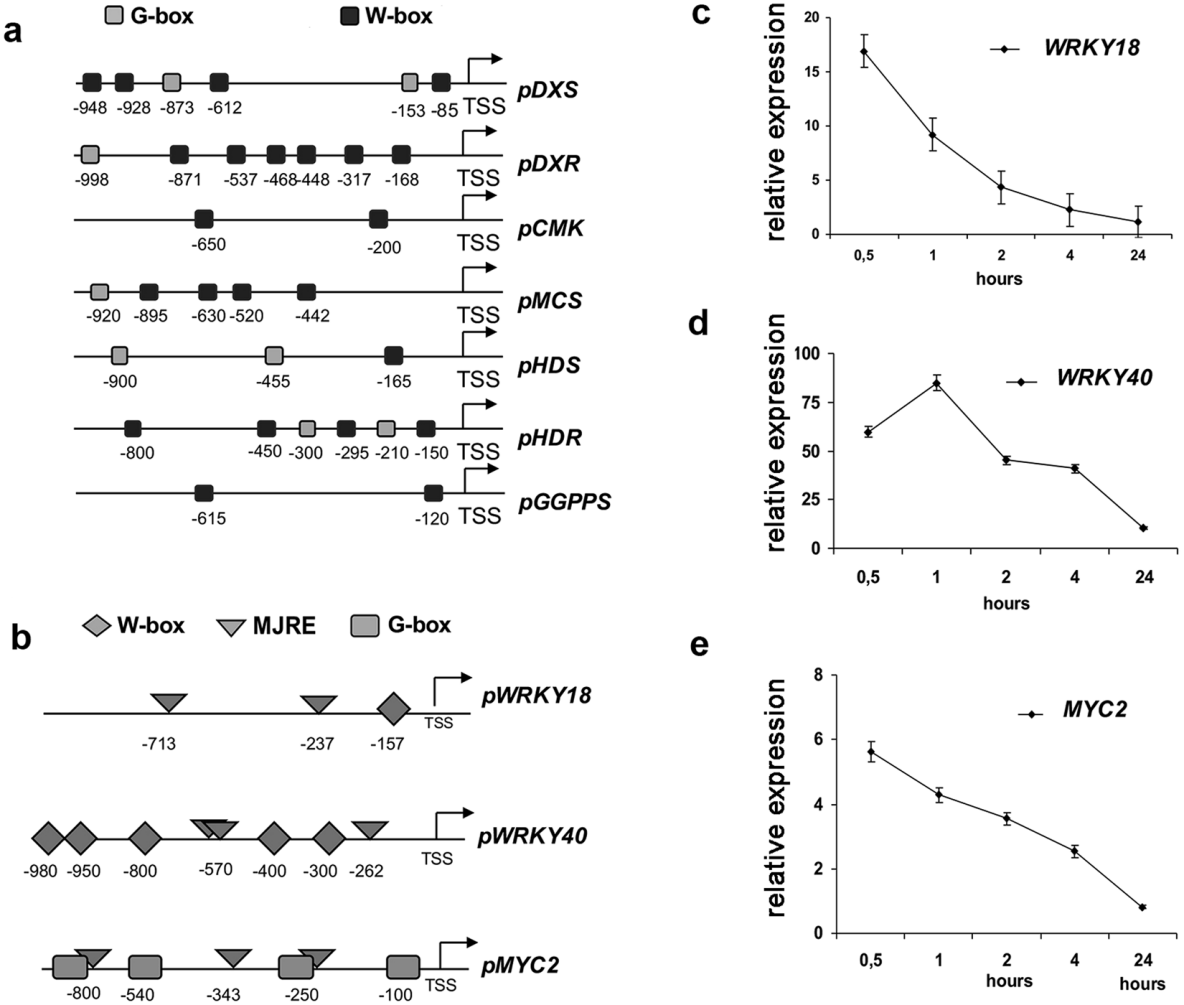
Identification and characterization of transcription factors likely involved in the regulation of genes encoding enzymes of the MEP-pathway in *A*. *thaliana*. Localization of representative W-box and G-box binding sites in the WRKYs and MYC2 promoters of *A*. *thaliana* MEP-pathway biosynthetic genes (**a**). Methyl-Jasmonate Responsive Elements (MJRE), W-box and G-box were present also in the 1000 bp region upstream the transcription start site (TSS) of the *AtMYC2*, *AtWRKY18* and *AtWRKY40* (**b**). Time–course transcriptional activation of *AtWRKY18* (**c**), *AtWRKY40* (**d**) and *AtMYC2* (**e**) TFs in *A*. *thaliana* seedlings in response to elicitation with MJ 150 µM for 24 h, measured by qRT-PCR and expressed as fold-changes compared to the expression level of untreated control seedlings. Values are means ± SD of three technical repeats from three biological replicates.

These data suggest a concerted gene regulation mediated by MJ, possibly through activations of TFs. To identify putative cis-regulatory DNA elements that might concert the MJ dependent activation of the MEP-pathway biosynthetic genes in *A*. *thaliana* a region of 1000 bp upstream the transcription start site point of *AtDXS*, *AtDXR*, *AtCMS*, *AtCMK*, *AtMCS*, *AtHDS*, *AtHDR*, *AtGPPS* genes was scanned by MatInspector software. W-box (*TTGAT/C*) and G-box (*CACGT*) binding sites of WRKY and MYC transcription factors, respectively, were found in the promoters of the analysed genes prevalently in multiple copies, conversely G-box were less abundant and not detected in *CMK* and *GGPPS* promoters (Fig. 3a). In particular, by using the online tool FIMO (MEME suite) for genome-wide promoter scanning analysis, a significant enrichment of the W-box *TTGAT/C* motifs was detected in the promoters of the MEP-pathway genes (Table S2) and a slight frequency increase of few G-boxes (Table S3). Interestingly, *cis*-acting elements involved in the MJ-responsiveness as well as W-boxes and G-boxes were found also in the promoter of *AtWRKY18*, *AtWRKY40*, and *AtMYC2* gene (Fig. 3b).

Altogether these data predict a potential involvement of MYC2 and members of WRKY TFs in the transcriptional regulation of biosynthetic genes of the MEP-derived terpene pathway in this species.

To gain more information about the expression profile of these TFs in response to MJ elicitation, we measured their transcript levels in two-week-old *A*. *thaliana* seedlings exposed to 150 μM MJ for 24 h or an equal volume of DMSO (mock control). The expression of *AtWRKY18*, *AtWRKY40*, and *AtMYC2* was strongly induced by MJ as early as 30 minutes of treatment (Fig. 3c, d, e, respectively). Remarkably, *AtWRK40* showed the highest up-regulation (fold change > 80, 1 h post treatment). *AtWRKY18* was also strongly up-regulated (fold change >15, 30′ post treatment). The MJ treatment induced also a significant overexpression of *AtMYC2* (fold change > 5, 30′ post-treatment). The MJ-induced activation of the three TFs precedes that of the biosynthetic genes involved in the MEP-pathway and their early activation suggests a possible role of WRKYs and MYC2 in the upstream regulation of the MEP pathway. Based on the high conservation of these genes in the plant kingdom, *AtWRKY18*, *AtWRKY40*, and *AtMYC2* were overexpressed in *S*. *sclarea* HRs, to establish their potential role in activating transcriptionally genes of the MEP-pathway in this plant species and, eventually, to enhance the synthesis of bioactive abietane diterpenes.

### Transformation and overexpression of *At*WRKY18, *At*WRKY40 and *At*MYC2 TFs in *S*. *sclarea* HR lines

**Figure 4:**
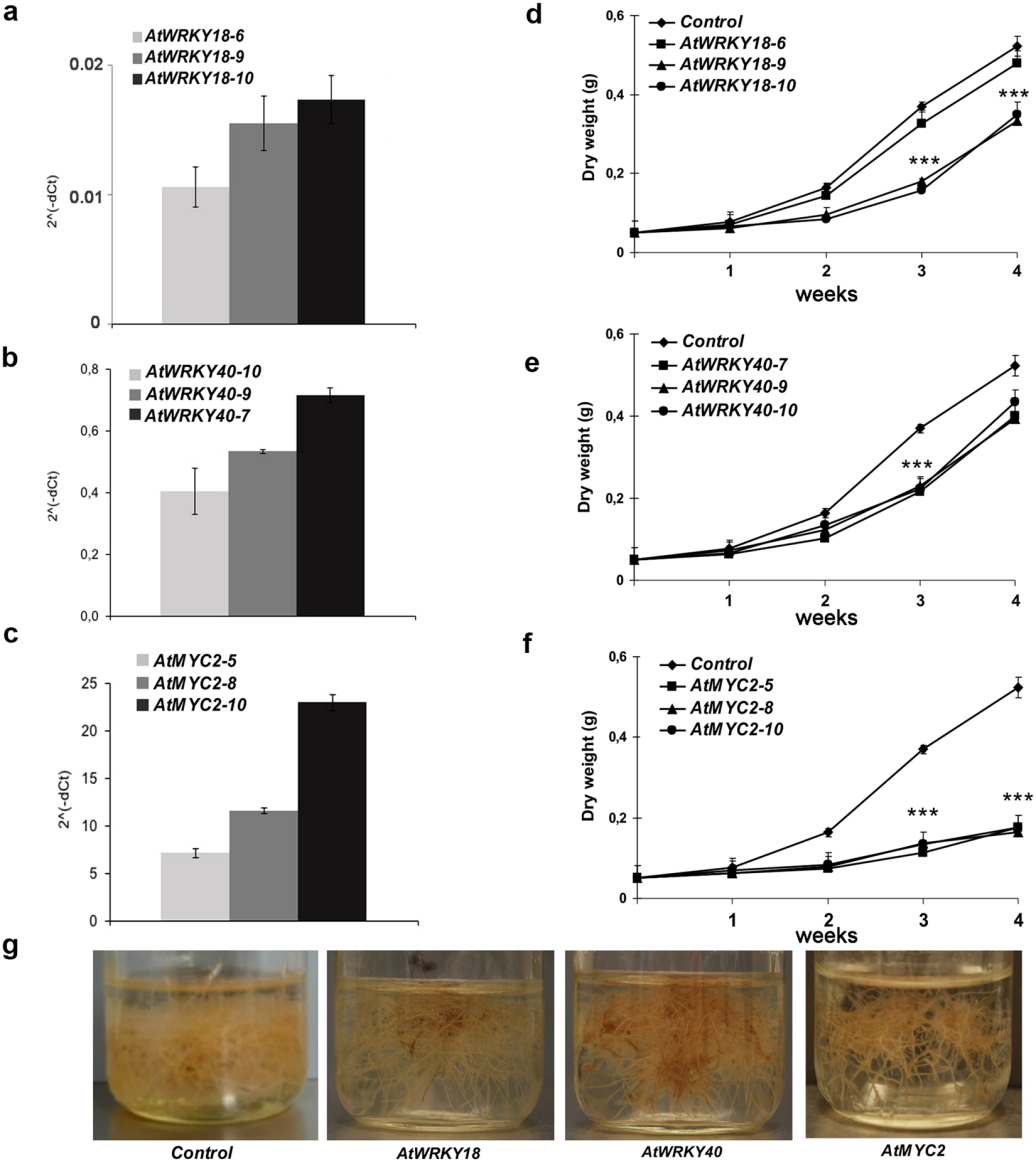
Expression levels of *AtWRKY18* (**a**) or *AtWRKY40* (**b)** or *AtMYC2* (**c**) in transgenic *S*. *sclarea* HR lines measured by qRT-PCR. 18S rRNA was used to normalize the expression of TFs. Values are reported as 2^−ΔCt^. Data represent mean values ± SD of three independent HR lines for each overexpressed TF, obtained from three technical replicates. Biomass production (mg g^−1^ hairy root dry weight) of three different *AtWRKY18* (**d**) or *AtWRKY40* (**e**) or *AtMYC2* (**f**) overexpressing HR lines compared to control HR line during 4 weeks of culture are also reported. The asterisks indicate a significant difference between control and transgenic HRs (P < 0.001) according to two-way ANOVA with Bonferroni post hoc test. Phenotypes of representative transgenic HR lines, grown in hormone-free liquid medium (**g**).

*S*. *sclarea* HRs overexpressing constitutively *AtWRKY18*, *AtWRKY40* or *AtMYC2* were generated by *Agrobacterium rhizogenes*-mediated transformation. Control lines were obtained by overexpressing a 174 bp fragment of *A*. *thaliana GUS* gene. Ten independent HR lines overexpressing each TF were obtained and kanamycin-resistant differentiated HRs were transferred into hormone-free medium and characterised for the expression of the transgenes, the absence of contaminating bacteria and the absence of any spurious amplification of endogenous TFs in the GUS control lines (Fig. S1a–d). Three independent HR lines overexpressing the three TFs were randomly selected for further experiments. The expression levels of the transgenes in the selected lines were determined by qRT-PCR (Fig. 4a–c).

To ascertain potential growth impairment associated to the TF overexpression, HR growth, measured as dry weight at interval of one week after the inoculation in fresh liquid medium, was also monitored for four weeks (Fig. 4d–f). All the overexpressing lines were characterised by a longer lag phase, particularly evident in the HR lines overexpressing the *At*MYC2 TF. The final dry weight was dependent on the level of expression of *AtWRKY18*: HR line #6, characterised by the lowest level of the transgene, showed a final biomass not significantly different from the control HR line. Overexpression of *At*WRKY40 did not cause any significant negative effect on the final HR growth, while a strong detrimental impairment was observed in all three HR lines overexpressing *At*MYC2. Independently of the effects on the HR growth, the overexpressing HR lines have an evident red colour compared to the control HR line (Fig. 4g), which indicates indirectly the accumulation of abietane diterpenes, as reported by Vaccaro *et al*.^3^.

### MEP-pathway biosynthetic genes are transcriptionally activated in *At*WRKY-18 and -40 and *At*MYC2 overexpressing *S*. *sclarea* hairy roots

To prove whether or not the three TFs control the biosynthetic genes involved in the synthesis of abietane diterpenes, the expression level of endogenous *SsDXS* (JZ903931.1), *SsDXR (*JZ903932.1), *SsCMK* (JZ903934.1), *SsHDS* (JZ903936.1), *SsGGPPS* (JZ903937.1) and *SsCPPS* (JZ903938.1) genes was measured by qRT-PCR in four week-old *S*. *sclarea* overexpressing HR lines, and compared with their level in the control HR line.

**Figure 5:**
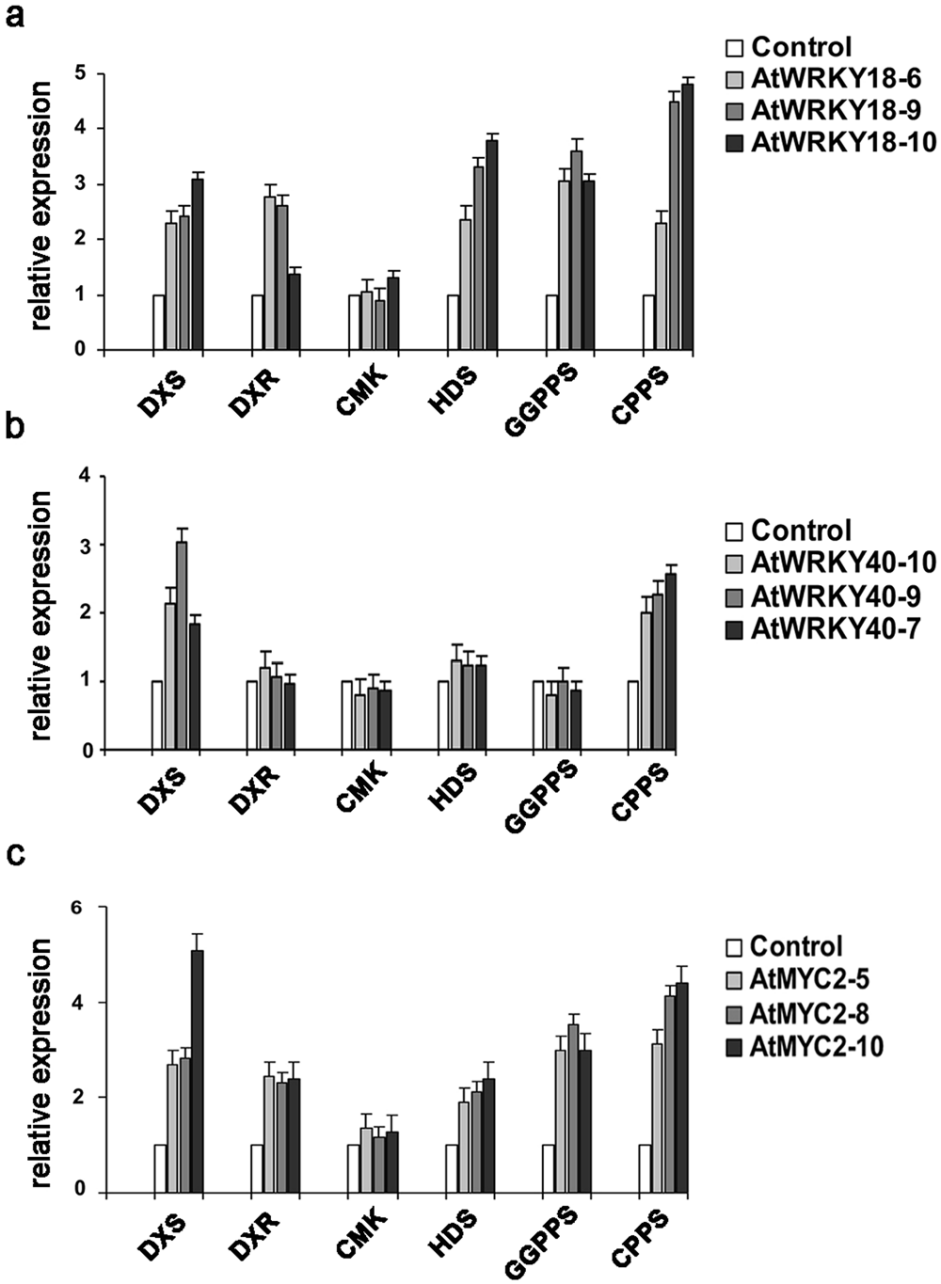
Relative expression level of MEP-pathway genes in *S*. *sclarea* HR lines overexpressing *AtWRKY18* (**a**), *AtWRKY40* (**b**) or *AtMYC2* (**c**) by qRT-PCR. *18S* rRNA was used to normalize the expression of TFs. Data represent mean ± SD of three technical repeats from three biological replicates, expressed as relative fold-changes compared to their basal level in control HR line.

Except for the *SsCMK*, all the analysed genes were up-regulated in *AtWRKY18* overexpressing HR lines, with a fold increase ranging from two to five (Fig. 5a). Interestingly, *SsCPPS*, encoding the first synthase responsible of conversion of GGPP into CPP, showed the highest expression level (about 5-fold increase) at least in two *AtWRKY18* HR lines. In *AtWRKY40* overexpressing lines, *SsDXS* (fold change > 3), encoding the first committed enzyme of the MEP-derived pathway, was preferentially up-regulated, together with *SsCPPS* (Fig. 5b). In all *AtMYC2* overexpressing HR lines, the expression levels of *SsDXS*, *SsDXR*, *SsHDS*, *SsGGPPS* and *SsCPPS* were notably increased, indicating that *AtMYC2* is a potent transcriptional activator of the MEP-pathway. Again, *SsDXS* (5-fold increase) and *SsCPPS* (more than 4-fold increase in the line *AtMYC2–10*) showed the highest transcriptional activation, while *SsCMK* transcript levels did not change significantly (Fig. 5c).

### Ectopic expression of *At*WRK-18, *-*40 and *At*MYC2 enhances the production of abietane diterpenes in HR lines

**Figure 6:**
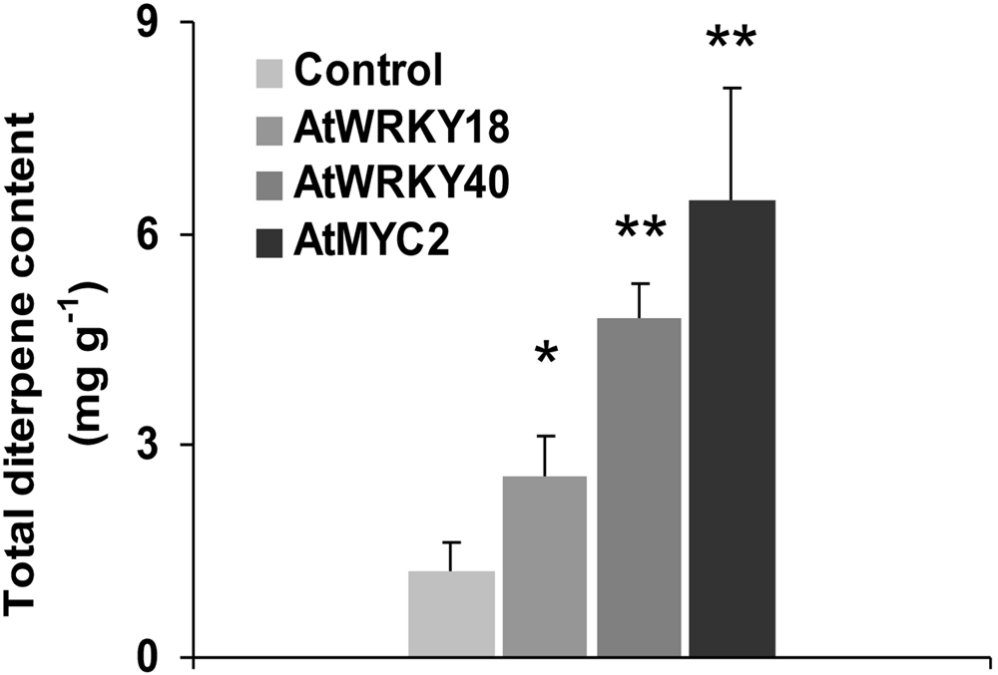
Total abietane diterpene content (mg g^−1^ dry weight), measured by HPLC-DAD analysis, in *AtWRKY18*, *AtWRKY40* and *AtMYC2* overexpressing *S*. *sclarea* HR lines. Values are means ± SD from three biological replicates and three technical repeats. Asterisks denote significant differences between the overexpressing HR lines and control HR lines (*P ≤ 0.05, **P ≤ 0.01, respectively).

The content of abietane derivatives was measured by targeted high-performance liquid chromatography with diode-array detector (HPLC-DAD) in acetone extracts obtained from *S*. *sclarea* HRs overexpressing the three TFs and compared to the content of the control HR line. Chemical structures of the most abundant *S*. *sclarea* abietane diterpenes and their typical chromatographic patterns are reported in Fig. S2, as comparison between the control and a representative transgenic line (*AtMYC2*, line #10). When examined collectively, the total abietane diterpene content varied with minimal fluctuations among the individual HR lines overexpressing each TFs overexpression (Fig. S3). *AtWRKY18*, *AtWRKY40* and *AtMYC2* boosted a significant increase in the total content of these compounds, expressed as mg g^−1^ dry weight (Fig. 6). Compared to the control HR line, *AtMYC2* overexpressing HR lines accumulated a significant higher content of abietanes (fold-increase > 5), followed by *At*WRKY40 overexpressing lines (fold-increase > 4) and by *AtWRK18* lines (fold-increase > 2) (Fig. 6 and Table S4).

**Figure 7:**
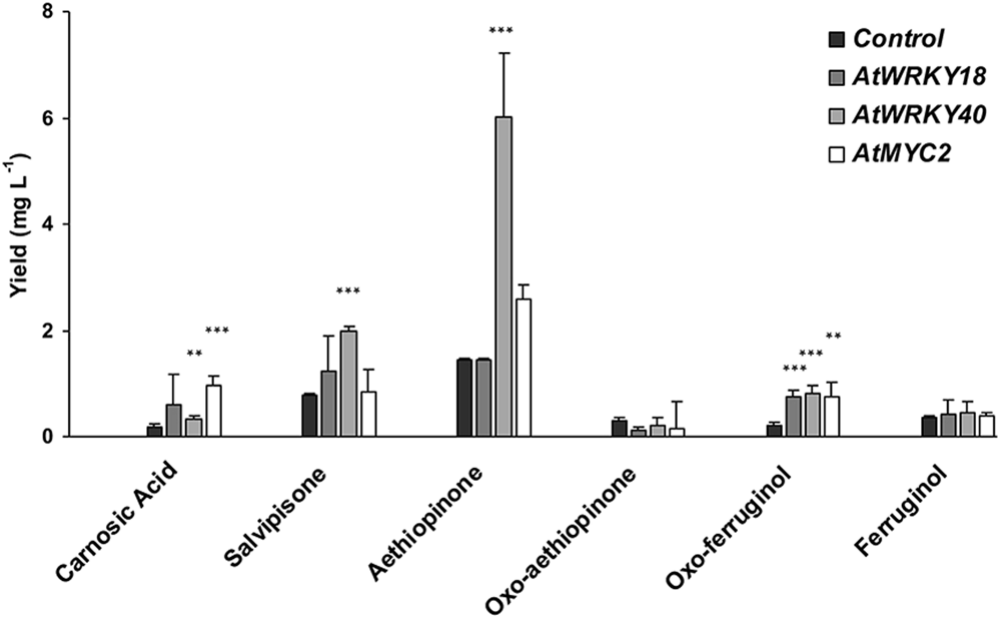
Final yield of abietanes diterpenes in *S*. *sclarea* HRs overexpressing the three different TFs. The yield was determined as amount of individual abietane compound (mg L^-1^) in the final biomass obtained from 1 liter of *AtWRKY18*, *AtWRKY40* and *AtMYC2* overexpressing *S*. *sclarea* HR lines. Values are means ± SD from three biological replicates. ** and *** indicate significant differences at P ≤ 0.01 and P ≤ 0.001, respectively, between the final yield of individual abietane diterpenes in the control and overexpressing HR lines.

However, we found that the ectopic expression of the three TFs affected in a variable manner the final HR growth (Fig. 4). Therefore, we also determined the final yield of individual abietane diterpenes in the different TF overexpressing HR lines (Fig. 7). A general increase in the final yield of carnosic acid, salvipisone and aethiopinone was obtained by overexpressing *AtWRK40*, which was not associated to a significant decrease in HR final dry weight (Fig. 4d) and preferentially activated the expression of the *DXS* and *CPPS* genes (Fig. 5b). Aethiopinone (>6 mg L^−1^) was the diterpene whose synthesis was highly enhanced (*P* < 0.001) by *At*WRK40 overexpression, with a 4.2 fold-increase above the basal level of the control HR line. As expected, the negative pleiotropic effect on final HR biomass due to the *At*MYC2 overexpression heavily penalized the final yield of individual abietane diterpenes.

## Discussion

Various biological activities have been reported for plant abietanes either of the abietane-phenolic type (*e*.*g*. carnosic acid and ferruginol) or abietane-quinone-type (aethiopinone, salvipisone and 1-oxoaethiopinone)^1,29^. Among them, aethiopinone has been shown to have anti-proliferative activity against HL-60 and NALM-6 leukemia cells^2^. We have also demonstrated that aethiopinone have an anti-proliferative activity in human melanoma cells, together with a safety profile for non-malignant cells^3^. Very low amount of these class pf diterpenes are synthesized in organs and tissues of several *Salvia* species and different biotechnological approaches (cell culture, hairy roots, elicitation) have been applied to enhance their content^3,5,30^. In the last few years, metabolic engineering strategies have been also attempted to increase the production of plant bioactive secondary metabolites, by tackling either single key-limiting genes or regulatory transcription factors, providing compelling evidence that it is feasible to boost the secondary metabolism in crop and medicinal plants^31–33^. Biosynthetic route of abietane diterpenes is tightly controlled by genes belonging to the well-conserved MEP-derived terpene pathway^34^. We have provided evidences that the content of abietane diterpenes can be enhanced by 2–3 times by overexpression of heterologous *AtDXS* and At*DXR* genes in *S*. *sclarea* HRs^5^ and more efficiently by MJ-elicitation (>20-fold increase), due to a coordinated transcriptional activation of several genes belonging to the plastidial MEP-derived terpene pathway^5^. These data indicate the existence of MJ-dependent transcriptional regulators of the MEP-pathway, as found for other metabolic pathways in several plant species^14,35^. It is well documented that metabolic engineering by targeting TFs is a powerful tool to manipulate the metabolic flux in plants and to eventually supersede the bottlenecks associated to targeting single genes^17,36,37^.

When we started our study on identifying putative regulatory TFs to boost the metabolic pathway of abietane diterpenes, information on the genome and transcriptome of *S*. *sclarea* and other *Salvia* spp were not publically available. Paralleling our previous results *S*. *sclarea*^5^, we found that biosynthetic genes of the MEP-pathway were transcriptionally activated by MJ in *A*. *thaliana* plantlets and that the promoter regions of *A*. *thaliana* MEP pathway genes contain G- and W-boxes, binding sites for MYC2 and WRKYs TFs. Noteworthy, in the case of W-boxes, hypergeometric distribution test revealed a significant enrichment of *TTGACT/C* motifs in the promoters of MEP pathway genes, corroborating the existence of a cooperatively regulation of these genes mediated by WRKY TFs in *A*. *thaliana*. Single or multiple copies of G-box and W-box were present in the promoter of the *GGPPS* gene, as already reported in the *S*. *milthiorriza* hortologous genes^38^, as well as in the promoter of genes encoding enzymes acting up-stream of GGPP. This suggests that WRKY and MYC TF members might exert a putative common regulatory mechanism of the MEP-pathway genes in plants. MYC2, a basic helix-loop-helix (bHLH) transcription factor, has been shown to be directly or indirectly involved in regulating secondary metabolites, through the MJ-induced degradation of the negative regulatory Jaz proteins, leading to the release of the *At*MYC2 and to the activation of its target genes biosynthesis^14^.

In the last few years WRKYs family is also emerging as another family of TFs controlling transcriptionally biosynthetic genes of metabolic pathways and as a valuable tool for enhancing the synthesis of different class of secondary metabolites^17^. For instance, *Cr*WRKY1 regulates the terpenoid indole alkaloide (TIA) biosynthesis by modulating the expression of several TIA genes in *Catharanthus roseus*^39^. *Ga*WRKY1 is a transcriptional activator of the gene *CAD1-A*, participating in cotton sesquiterpene biosynthesis^12^. Overexpression of *Tc*WRKY1 increases the expression of 10-deacetylbaccatin III-10 β-O-acetyl transferase (DBAT), which plays a prominent role in the biosynthesis of taxol in *Taxus chinensis*^40^. Interestingly, publically available Arabidopsis microarrays revealed that about 30% (22 of 72) of WRKY TFs respond to jasmonate treatments^21^. Remarkablly, a recent genome-wide binding analysis in *A*. *thaliana* have indicated that *At*WRKY18 and *At*WRKY40 bind the promoter of several target genes, known to be transcriptionally regulated in a MJ-dependent fashion^23^.

In this study, we have confirmed that *At*MYC2 and two WRKY TFs, *At*WRKY18 and *At*WRKY40, which contain MJ responsive elements (*G/ATGTT*) in their promoters, are transcriptionally regulated by MJ in Arabidopsis seedlings. In addition, W-box elements have been found in the promoters of AtWRKY18 and AtWRKY40 suggesting a possible autocatalytic regulation, in line with the well established auto- and cross-regulation mechanisms described for the WRKY family^16^. It has been also reported recently that several WRKY TFs are activated by MJ in *S*. *miltiorrhyza* HRs, which, in turn, accumulated high levels of tanshinones, a class of abietane-type diterpenoids^41^.

To establish their potential role in controlling the production of abietane diterpenes, *S*. *sclarea* HR lines overexpressing these three TFs were generated and we demonstrated that *AtWRKY18*, *AtWRKY40* and *AtMYC2* TFs were able to regulate the expression of several biosynthetic genes belonging to the plastidial MEP-derived pathway. AtWRKY18 activated significantly the expression of *S*. *sclarea* genes *DXS*, *DXR*, *HDS*, and *GPPS* genes, encoding enzymes involved in the synthesis GGPP, the universal precursor of diterpenes and also the expression level of the CPPS gene, encoding a synthase converting the cyclization of GGPP into copalyl diphosphate (CPP). It has been reported that CPP is the precursor of ferruginol and carnosic acid^42,43^, two abietane diterpenes, and might be the precursor also of aethiopinone, the main abietane diterpene found in *S*. *sclarea* roots, although this has to be further proved. Instead, overexpression of *At*WRK40 was able to activate preferentially the transcription of *DXS* gene, encoding the first committed step of the MEP-pathway and the *CPPS* gene. Further studies are necessary to elucidate whether the transcriptionally activation of genes encoding enzymes of the MEP-pathway is due to a direct binding of these two WRKY TFs to the promoters of the targeted genes or through activation of up-streams TFs. However, in all the *AtWRKY-18* and *-40* overexpressing HR lines, the level of transcriptional activation of the MEP biosynthetic genes was far below the fold-increase that we have reported by MJ treatment^5^. This might be related to the occurrence of a concerted transcriptional activation of the whole metabolic pathway not necessarily guided by the action of a single TF, but rather by a combinatorial role for several TFs^44^. Actually, it has been reported that *At*WRKY18 and *At*WRK40 interact physically and functionally with each other to form homo- and heterodimers^22^, indirectly suggesting that these WRKY TFs may act in an additive or synergistic manner to drive metabolic flux towards specific compounds. Owing to their closely related functions, it would be interesting to ascertain the possibility to control the metabolic pathway of abietane diterpenes by co-expression of both these WRKY TFs in *S*. *sclarea* HRs.

Biosynthetic genes encoding DXS, DXR, HDS, and GGPPS enzymes were also transcriptionally regulated in *S*. *sclarea* HR by overexpressing *At*MYC2, in a quantitative manner as found in *At*WRK18 overexpressing HR lines. This confirms MYC2 as a master regulatory factor in the MJ activation of the secondary metabolism, possibly by controlling other TFs or biosynthetic genes. In *A*. *thaliana* studies on *At*MYC2 have been primarily focused on establishing its role in mediating the overall transcriptional changes in response to biotic and abiotic stresses^15^. However, a wealth of recent information is also uncovering the role of MYC2 in regulating specifically biosynthetic genes. In *Catharanthus roseus*, *Cr*MYC2 regulates the expression of ORCA2 and ORCA3 TFs, which, in turn, tune the expression of alkaloid biosynthetic genes through a MJ- dependent manner^45^. Tobacco bHLH1 and bHLH2 TFs, similar to *At*MYC2 and *Cr*MYC2, were functionally identified as regulators of nicotine biosynthesis using virus-induced silencing in MJ-*treated N*. *benthamiana*, by decreasing the expression of PMT gene (putrescine *N*-methyltransferase) and MPO gene (N-methylpitrescine oxidase) and overexpression of *N*. *tabacum* counterpart genes also cooperatively activated the PMT2 gene^46,47^. *AaMYC2* overexpression significantly activated the transcript levels of *CYP71AV1* and *DBR2*, which resulted in an increased artemisinin and anthocyanin content in *A*. *annua*^27^. Recently, the regulatory role of MYC TFs have been proved in *Salvia miltiorrhyza*: *SmMYC2a* and *SmMYC2b*, which are however divergent from *AtMYC2*, are positive regulators of *SmPAL*, *SmHPPR*, *SmHCT6*, *SmCYP98A14*, *SmMK*, *SmCMK*, *SmMCS*, *SmCPS* and *SmKSL*, encoding enzymes of the biosynthetic route of tanshinones and phenolic acids in the roots of this species^48^.

Although the three Arabidopsis TFs were able to activate transcriptionally genes encoding enzymes acting upstream the GGPP as well as the *CPPS* gene in *S*. *sclarea* HRs, it remains to ascertain whether the observed transcriptional regulation is due to a direct binding of W- box and G-box present in the promoters of these biosynthetic genes and/or by activating other TFs, as demonstrated for *At*MYC2^15^.

The transcriptional up-regulation of biosynthetic genes by *At*MYC2, *At*WRKY18 and *At*WRK40 overexpression was associated to a general increased accumulation of total abietane diterpenes, compared to the control HR line. Considering the total abietane diterpenes content, the most boosting effect was obtained by overexpressing *At*MYC2, which induced a coordinated up-regulation of four out of the five analysed biosynthetic genes belonging to the MEP-derived pathway as well as of *CPPS* gene. The content of aethiopinone, the compound for which we have reported a novel anti-proliferative activity against melanoma cells^3^, was enhanced consistently (8-fold increase) in the HR line with the highest transcript level of *At*MYC2. Surprisingly, the same enhancing effect on the content of abietane diterpenes was not triggered by overexpressing *At*WRKY18, which was shown to induce transcriptionally the same group of biosynthetic genes. We can only speculate that in *At*WRKY18 overexpressing HR lines the ratio between precursor content and enzyme level in each step of this metabolic pathway was somehow unbalanced, preventing an expected accumulation of abietane diterpenes. This aspect has a great relevance in designing production platforms of secondary metabolites based on metabolic engineering^49^. Interestingly, the overexpression of *At*WRKY40 in *S*. *sclarea* HR lines, which activated almost preferentially the activation of the *DXS* and *CPPS* genes, was sufficient to boost a significant accumulation of abietane diterpenes, especially aethiopinone and salvipisone content (6 and 3 fold-increase, respectively). These data confirm our previous findings evidencing that DXS and CPPS are limiting enzymes for the synthesis of abietane diterpenes in *S. sclarea* HR lines^3,5^.

Another aspect of great relevance in metabolic engineering is that a single TF may be involved in multiple molecular response and cross-talk among different metabolic pathways. The overexpression of a TF might have a strong negative effect on plant cell, organ and tissue growth and final biomass, as reported for overexpression of ORCA3 in *C*. *roseus*^50^. Although *AtMYC2* overexpressing HR lines accumulated the highest content of each abietane compound, the growth of these transgenic HR was drastically impaired, affecting negatively the final yield of this class of compounds. This is not surprisingly given the multitasking role of MYC2 as a point of crosstalk between distinct signalling cascades, including light, abscisic acid and jasmonic acid^51^. Possible negative effects of constitutive *At*MYC2 overexpression could be circumvented, using an inducible promoter to drive its expression. In our hands, the overexpression of *At*WRK40 induced the highest final yield in abietane diterpenes, especially aethiopinone, due to a trade-off between the expression level of biosynthetic genes and HR growth.

In conclusion, the data presented herewith demonstrated the feasibility of enhancing the content of abietane diterpenes by ectopic expression of heterologous Arabidopsis TFs, which were able to regulate in a coordinated manner the expression of several genes of the MEP-derived terpene pathway and the *CPPS* gene. To our knowledge, this is the first example of metabolic engineering of abietane diterpenes in *S*. *sclarea* using WRKY TFs. The present results are also informative and instrumental to enhance the synthesis of abietane diterpenes derived from the plastidial MEP-derived terpenoid pathway in other *Salvia* species.

## Material and Treatments

### Plant material, growth conditions and treatments

*A*. *thaliana* (Columbia ecotype) seeds were surface sterilised with 70% (v/v) ethanol for 1 min and then in 2% (v/v) sodium hypochlorite solution for 10 min, thoroughly washed, stratified for 48 h at 4 °C in the dark and sown on Murashige & Skoog^52^ solid medium and grown at 23 °C, under long-day photoperiod (16 h light, 8 h dark) in presence of cool white fluorescent light (110 μmol m^−2^ s^−1^). Groups of 25 two-week-old seedlings were transferred in MS liquid medium, according to Guo *et al*.^53^. After seven days, seedlings were treated for 0.5, 1, 2, 4 and 24 h with 150 μM MJ (Sigma, Saint Louis, USA) under the same temperature and photoperiod conditions described above. The experiments were carried in three biological replicates.

*S*. *sclarea* hairy roots were obtained from axenic plants by transformation with *A*. *rhizogenes* ATCC15834, as previously reported in Vaccaro *et al*.^3^, and sub-cultured in MS hormone-free medium for four weeks. *S*. *sclarea* HR were treated for seven days with 150 µM Methyl-jasmonate (MJ) or an equal volume of DMSO, as mock control.

Tissue samples were collected at different time intervals from control or MJ-treated *A*. *thaliana* plantlets and *S*. *sclarea* HRs, immediately frozen and kept at −80 °C for gene expression and metabolic analyses.

### Bioinformatic analysis

The 1000 bp sequences upstream the Transcription Starting Site of *A*. *thaliana WRKY18* (AT4G31800), *WRKY40* (AT1G80840), *MYC2* (AT1G32640), *DXS* (AT4G15560), *DXR* (AT5G62790), *CMK* (AT2G26930), *MCS* (AT1G63970), *HDS* (AT5G60600) *and GGPPS* (AT1G49530), were retrieved by the National Center for Biotechnology Information (NCBI) database (http://www.ncbi.nih.nim.gov/) and scanned by Genomatix Matinspector professional (http://www.genomatix.de/cgi-bin/matinspector_prof/mat_fam.p1), to identify putative cis-regulatory sequences.

Genome-wide scanning of G-box and W-box motifs was carried out by the online tool FIMO (Find Individual Motif Occurrences)^54^. The genomic *Arabidopsis* promoter dataset containing 37694 sequences (approximatively 1000 bp upstream the transcription starting site) was obtained from Genomatix (version *TAIR10_1)*. Promoter Motif scanning was carried out by searching for MYC and WRKY core motifs and accepted variants as reported by Godoy et^55^ and Eulgem *et al*.^56^, respectively. Data were expressed as number of a given cis-element per promoter (cis-element frequency) and percentage of promoters with a given cis-element.

### Plasmid construction, HR transformation and growth analysis

The plasmids containing the full-length cDNA of *A*. *thaliana WRKY18* (Accession n. U14890), *WRKY40* (Accession n. C105126) e *MYC2* (Accession n. U12679) were obtained from the *Arabidopsis Biological Resource Center* (https://abrc.osu.edu). The coding sequences of the genes were amplified by polymerase chain reaction (PCR) using a *High-Fidelity* DNA Polymerase (*Pfx*, Invitrogen, Carlsbad, CA, USA) with specific primers (Table S5), characterised by the presence of the short sequence *CACC* at 5′ for the cloning in pENTR/ kit D-TOPO® (Invitrogen) to generate an *Entry-Clone*. The correct insertion and the absence of mutations were verified by sequencing (Primm Biotech, Milan, Italy). Subsequently, coding sequences of the transcription factors were subcloned through the LR reaction (*The Gateway® LR Clonase™ enzyme mix kit*, *Invitrogen*) in the gateway *Destination vector* pGBW17 (kindly provided by Prof. Nakagawa^57^, driven by the constitutive strong viral 35SCaMV promoter, and containing the C-terminal 4xmyc-tag and kanamycin resistance selectable marker^58^, through a site-specific recombination^59^. The resulting binary vectors were mobilised into the *A*. *rhizogenes* ATCC 15834, by the standard freeze/thaw cycle and CaCl_2_ method^60^ and used to transform leaf disks from 20 day-old *S*. *sclarea* axenic plantlets, as previously reported^3^. After several transfers to medium containing 50 mg l^−1^ kanamycin and decreasing concentrations of cefotaxime (from 100 down to 50 mg l^−1^), HRs developing from the infected areas were individually excised, sub-cultured several times on hormone-free medium and maintained at 23 °C in the dark. Kanamycin HR independent lines, with no bacterial contamination, were selected, and sub-cultured into 250 ml flasks, containing a hormone-free liquid MS medium supplemented with 50 mg l^−1^ kanamycin and kept on a gyratory shaker at 120 rpm at 23 °C in the dark. The HR lines were sub-cultured every week.

HR growth was analysed by inoculating equal amounts (0.5 g) of untransformed or TF overexpressing HR lines into MS hormone-free liquid medium. Dry weight was monitored during one month at one-week intervals.

### Nucleic acid purification, PCR and RT-PCR analyses

Genomic DNA was extracted from HR lines by the cetryl trimethyl ammonium bromide (CTAB) method as described in Doyle and Doyle^61^ and used as template in PCR reactions to establish stable integration of *AtMYC2*, *AtWRKY18* and *AtWRKY40* in transgenic HRs, using a forward primer located at the promoter of the PGBW17:35S^2^ and a reverse primer located at the 3′ end of the respective genes. Amplification reactions were set using 2.5 units of Taq polymerase (Invitrogen, Carlsbad, CA, USA), and run as follows: 94 °C for 5 min, followed by 30 cycles at 94 °C 30 sec, annealing 30 sec, at the different T_m_, according to the primers melting temperatures, 72 °C 1 min, and a final extension 72 °C for 5 min. To detect the absence of contaminating *Agrobacterium*, the HR genomic DNA from control and transformed HRs was used as template to amplify the *virD2* gene, using specific primers^62^.

Total RNA, from *A*. *thaliana* plantlets or *S*. *sclarea* HRs, was extracted using the plant RNA/DNA Purification kit (Norgen Biotek Corporation Ontario, Canada), according to the manufacturer’s protocol. For semi-quantitative RT-PCR, complementary DNA was synthesized from 1 μg total RNA, previously treated with RNase-free DNAse I, using random hexamers and the Superscript III RT (Invitrogen, Carlsbad, CA, USA) at 50 °C for 50 min. In the PCR reactions one microliter of cDNA was used as template with specific primers (Table S5) and 2.5 units of Taq polymerase (Invitrogen, Carlsbad, CA, USA), using the following conditions: initial denaturation at 94 °C 1 min, followed by 30 cycles denaturation at 94 °C 30 sec, annealing 30 sec at the different T_*m*_, according to the primers melting temperatures, extension at 72 °C for a time depending on the length of the DNA target and final extension at 72 °C for 5 min.

For qRT-PCR, complementary DNA was synthesized as described above. The reactions were performed in a 20 µl volume, containing several cDNA dilutions and 0.5 µM primers, specific for each gene. Cycler-DNA Master SYBR Green I mix (Roche Diagnostics Ltd, Lewes, UK). The reactions were run in a Light Cycler rapid thermal cycler system (Roche Diagnostics Ltd, Lewes, UK) under the following fast cycling steps: initial denaturation for 2 min at 94 °C, followed by 40 cycles at 94 °C for 2 s, 58 °C for 30 s. In addition, melting curves (20 min; from 58 °C to 90 °C) were generated to check any spurious amplification products. To normalize RNA levels, *S*. *sclarea* or *A*. *thaliana 18S* rRNA was used as internal reference control. The sequences of all the primers used are listed in Table S5. At least three technical repeats from three biological replicates were carried out. Level of gene expression of the three exogenous TFs and of endogenous biosynthetic genes of the MEP-pathway in different overexpressing hairy root lines and the control lines were represented as 2^-deltaCt ^^63^.

### Qualitative and quantitative determination of abietane diterpenes

Abietane diterpenes were identified and quantified as previously reported^3,5^. Briefly, lyophilized and powdered hairy roots (0.5 g) were extracted with acetone for 72 h at room temperature and the obtained residues dissolved in methanol and subjected to HPLC-DAD analysis (Agilent1200 Series, G1312A binary pump, G1329A automatic sample injector, G1315D diode array detector). The HPLC fingerprint was carried out on a C_8_ column (Agilent, Zorbax eclips C_8_ 250 × 4.6 mm) with a sample injection volume of 50 µl. The mobile phase was a gradient elution of water acidified with 0.1% formic acid (solvent A) and acetonitrile (solvent B), starting with 35% B and rising to 100% B after 30 min, at a flow rate of 1.0 ml min^−1^. The different diterpenes were detected at 280 nm by comparing with standard purified compounds and concentration calculated by the interpolation of the peak areas with calibration curves, constructed over the range 10–200 µg ml^−1^ of purified compound. Content of diterpenoids in roots was expressed as mg g^−1^ of HR dry weight.

### Statistical analysis

All data are represented as mean ± SD of at least three independent biological experiments performed in triplicate. The statistical significance of HR growth rate and diterpene content values was established by the two-way analysis of variance, with Bonferroni post-hoc test analysis, using the GraphPad Prism 5 software. Differences between control and transgenic HR lines were considered to be significant at least when *P* < 0.05.

Statistical analyses for discovering the enrichment of W box and G cis-element sequences in the promoters of Arabidopsis MEP pathway genes were performed by Hypergeometric test^64^. The test was performed by counting and comparing the number of the promoters of MEP pathway genes containing a given G- and W-box motif (number of success) with respect to the all Arabidopsis promoters (number of success in the population).

### Data availability

All data generated or analysed during this study are included in this published article and its Supplementary Information files.

## Electronic supplementary material

Supplementary materials
