# Supplementary material for: Coactivation of MEP-biosynthetic genes and accumulation of abietane diterpenes in Salvia sclarea by heterologous expression of WRKY and MYC2 transcription factors
Source: Sci Rep. 2018 Jul 20;8:11009. doi: 10.1038/s41598-018-29389-4 (PMC6054658; doi:10.1038/s41598-018-29389-4)
Supplement: Supplementary file 1 — Supplementary materials [file 41598_2018_29389_MOESM1_ESM.docx]

**Coactivation of MEP*-*biosynthetic genes and accumulation of abietane diterpenes in *Salvia sclarea* by heterologous expression of WRKY and MYC2 transcription factors**

Mariaevelina Alfieri^1^, Maria Carmela Vaccaro^1^, Elisa Cappetta^1#^, Alfredo Ambrosone^1^, Nunziatina De Tommasi^1^, Leone Antonietta^1^*

1) Department of Pharmacy, University of Salerno, Via Giovanni Paolo II 134D, 80084 Fisciano, Italy

*corresponding author: Antonietta Leone

Email: [aleone@unisa.it](mailto:claudia.tortiglione@cnr.it)

1) Department of Pharmacy, University of Salerno, Via Giovanni Paolo II 134D, 80084 Fisciano, Italy

* corresponding author: Antonietta Leone

Email: [aleone@unisa.it](mailto:claudia.tortiglione@cnr.it)

# current address: Department of Agricultural Sciences, University of Naples Federico II, via Università 100, 80055 Portici (Naples) Italy


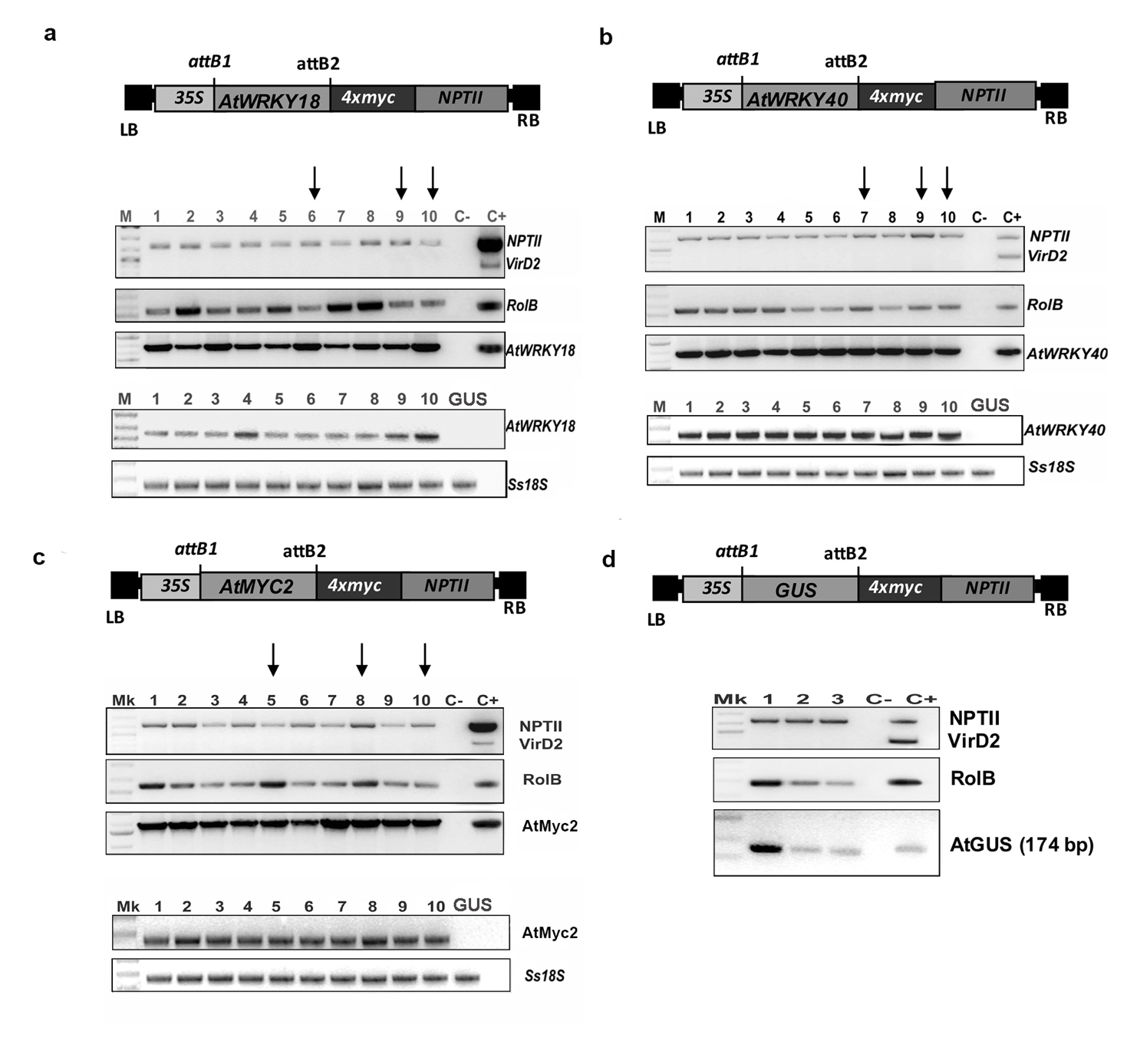


**Figure S1.** Molecular characterization of *AtWRKY18* (a) *or AtWRKY40* (b) *or AtMYC2* (c) or *GUS* (d) overexpressing hairy root lines. For each transgene is reported the schematic representation of the plasmid construct used for the constitutive overexpression. LB, left border; RB, right border; attb1 and attb2, homologous recombination sites; PCR amplification of genomic DNA using specific primers for *NPTII*, *rolB* and *AtWRKY18* or *WRKY40* or *MYC2* or *GUS* genes. Genomic DNA was also used to amplify the *VirD2* gene to confirm the absence of contaminant bacteria. M, marker; C-, negative control without DNA sample; C+, *A. rhizogenes* plasmid carrying the exogenous gene. Semi-quantitative RT-PCR showing the expression of the exogenous *AtWRKY18* or *WRKY40* or *MYC2* transcripts in all transgenic hairy root lines. GUS, Hairy root lines transformed with GUS vector.

Arrows denote HR selected for further characterization. Cropped images retain all the bands available on each gel electrophoresis.


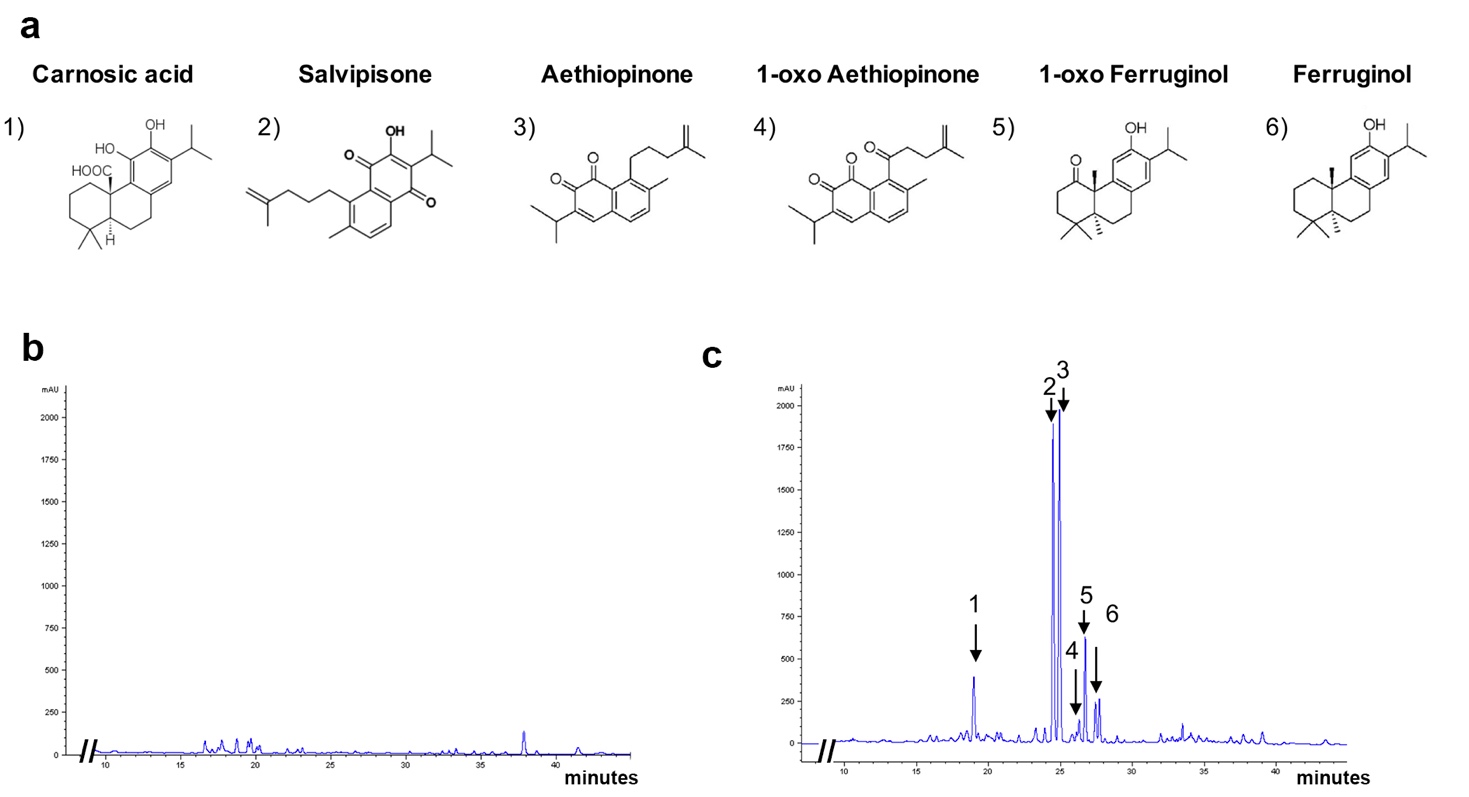


**Figure S2.** Chemical structures of principal abietane diterpenes synthetised in the roots of *Salvia sclarea* (a, upper panel). Comparison of the chromatograms of control hairy root line (b) with a representative hairy root line (*AtMYC2#5)* (c).

**
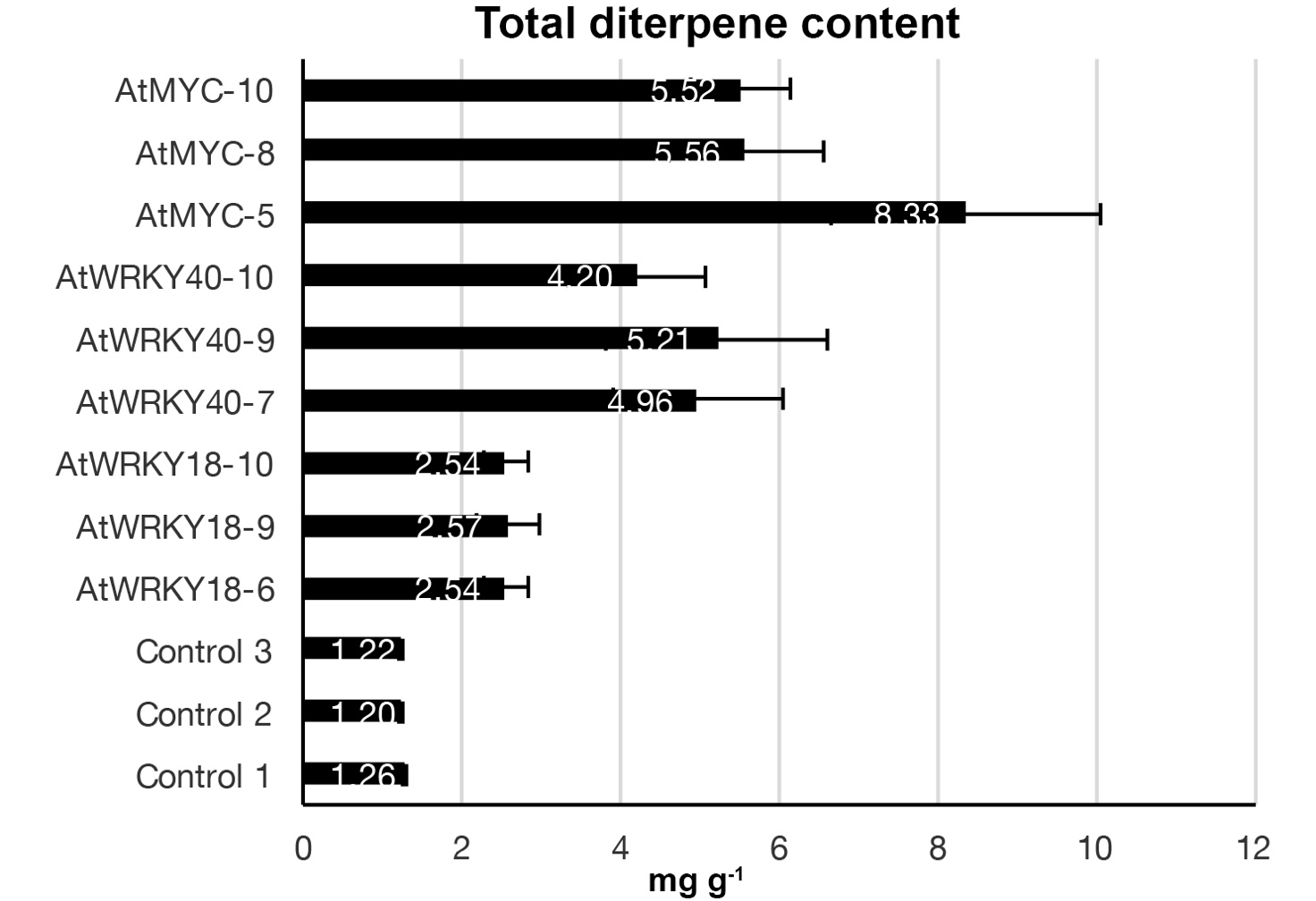
**

**Figure S3. Total abietane diterpene content (mg g^-1^).** The content of abietanes was measured by HPLC-DAD analysis in control and TF overexpressing hairy roots of *S. sclarea.*

|  | ***Salvia sclarea*** |  | ***Arabidopsis thaliana*** | |
| --- | --- | --- | --- | --- |
| **genes** | **6 h** | **24 h** | **6 h** | **24 h** |
| *DXS* | 6.3 ± 1.03 *** | 56.43 ± 1.57*** | 7.52 ± 0.33*** | 2.89 ± 0.22** |
| *DXR* | 17.27 ± 1.27*** | 55.04 ± 1.63*** | 10.13 ± 0.50*** | 3.53 ± 0.37** |
| *CMS* | 1.44 ± 0.26 | 6.44 ± 0.68*** | 4.42 ± 0.25*** | 2.55 ± 0.25** |
| *CMK* | 1.06 ± 0.57 | 2.63 ± 0.66** | 2.2 ± 0.12* | 1.9 ± 0.13* |
| *MCS* | 1.79 ± 0.35* | 4.3 ± 0.66*** | 4.86 ± 0.54*** | 3.08 ± 0.43** |
| *HDS* | 8.60 ± 1.22*** | 35.00 ± 1.63*** | 5.98 ± 0.23*** | 3.64 ± 0.15** |
| *GGPPS* | 7.56 ± 1.23*** | 23.86 ± 1.68*** | 7.31 ± 0.27*** | 3.81 ± 0.34** |

**Table S1.** Expression levels of MEP-pathway genes by qRT-PCR upon 150 uM MJ elicitation in *S. sclarea* and *A. thaliana*. *, ** and *** indicate significant differences at P≤0.05, P≤0.01 and P≤0.001, respectively.

|  | **All Arabidopsis promoters** | | **MEP pathway promoters** | |
| --- | --- | --- | --- | --- |
|  | **% promoters with the element** | **Cis-element frequency** | **% promoters with the element** | **Cis-element frequency** |
| TTGACT/C | 46,86 | 0,72 | 85,71* | 0,71 |
| TGACT | 70,60 | 1,03 | 85,71 | 1,57 |
| TTTGAC | 38,42 | 0,47 | 71,43 | 0,71 |
| TTGAC | 69,91 | 1,27 | 85,71 | 1,71 |
|  |  |  |  |  |

**Table S2.** Enrichment analysis of cis-elements related to WRKY TFs. Data were represented as i) percentage of promoters containing distinct elements and ii) cis-element frequency expressed as the number of motifs per promoter. Asterisk denotes a significant difference between the percentage of promoters containing the cis-element according to the Hypergeometric distribution (p<0.05)

|  | **All Arabidopsis promoters** | | **MEP pathway promoters** | |
| --- | --- | --- | --- | --- |
|  | **% promoters with the element** | **Cis-element frequency** | **% promoters with the element** | **Cis-element frequency** |
| CACGTG | 16,15 | 0,20 | 14,29 | 0,14 |
| AACGTG | 16,14 | 0,14 | 14,29 | 0,16 |
| CATGTG | 23,61 | 0,40 | 42,86 | 0,57 |
| CAGCTG | 12,65 | 0,14 | 14,29 | 0,14 |
| CAAGTG | 17,50 | 0,19 | 42,86 | 0,43 |
| CAAATG | 37,98 | 0,49 | 71,43 | 0,71 |

**Table S3.** Enrichment analysis of cis-elements related to MYC TFs in the promoters of MEP pathway genes. Data were represented as i) percentage of promoters containing distinct elements and ii) cis-element frequency expressed as the number of motifs per promoter.

| **Transgenic root line** | **Carnosic Acid** | **Salvipisone** | **Aethiopinone** | **Oxo-aethiopinone** | **OxoFerruginol** | **Ferruginol** | **Fold increase (Abietanes total content )** |
| --- | --- | --- | --- | --- | --- | --- | --- |
|  |  |  |  |  |  |  |  |
| ***Control*** | 0.073 ± 0.001 | 0.283 ± 0.009 | 0.524 ± 0.015 | 0.136 ± 0.007 | 0.080 ± 0.003 | 0.131 ± 0.002 | **1** |
| ***AtWRKY18-6*** | 0.572 ± 0.007 | 0.691 ± 0.016 | 0.738 ± 0.027 | 0.043 ± 0.007 | 0.319 ± 0.011 | 0.175 ± 0.004 | **2.08** |
| ***AtWRKY18-9*** | 0.174 ± 0.084 | 1.025 ± 0.076 | 0.616 ± 0.017 | 0.035 ± 0.002 | 0.578 ± 0.006 | 0.136 ± 0.003 | **2.09** |
| ***AtWRKY18-10*** | 0.182 ± 0.032 | 0.299 ± 0.019 | 0.978 ± 0.007 | 0.183 ± 0.005 | 0.443 ± 0.041 | 0.451 ± 0.002 | **2.07** |
| ***AtWRKY40-7*** | 0.215 ± 0.014 | 0.991 ± 0.006 | 2.921 ± 0.007 | 0.027 ± 0.003 | 0.507 ± 0.006 | 0.299 ± 0.007 | **4.06** |
| ***AtWRKY40-9*** | 0.176 ± 0.019 | 0.466 ± 0.048 | 3.697 ± 0.319 | 0.109 ± 0.013 | 0.424 ± 0.048 | 0.339 ± 0.031 | **4.27** |
| ***AtWRKY40-10*** | 0.135 ± 0.005 | 1.074 ± 0.004 | 2.324 ± 0.054 | 0.181± 0.002 | 0.341 ± 0.015 | 0.146 ± 0.003 | **3.44** |
| ***AtMYC2-5*** | 1.331 ± 0.078 | 0.742 ± 0.008 | 4.782 ± 0.198 | 0.361 ± 0.007 | 0.593 ± 0.006 | 0.515 ± 0.005 | **6.81** |
| ***AtMYC2-8*** | 1.146 ± 0.024 | 0.762 ± 0.012 | 2.778 ± 0.07 | 0.045 ± 0.003 | 0.391± 0.009 | 0.437 ± 0.015 | **4.55** |
| ***AtMYC2-10*** | 0.700 ± 0.027 | 1.558± 0.05 | 1.782 ± 0.042 | 0.103 ± 0.013 | 0.800 ± 0.022 | 0.572 ± 0.017 | **4.51** |
|  |  |  |  |  |  |  |  |

**Table S4.** Total abietane diterpene content (mg g^-1^), measured by HPLC-DAD analysis, produced in the three selected *AtWRKY18, AtWRKY40* and At*Myc2* overexpressing *S. sclarea* hairy roots compared to control root lines.

| Name | Accession number | Sequence 5’- 3’ | Use |
| --- | --- | --- | --- |
| WRKY18 | AT4G31800 | Fw- CACCATGGACGGTTCTTCGTTTCT | Cloning into pEntr-D-TOPO |
|  |  | Rev- TGTTCTAGATTGCTCCATTA |  |
| WRKY40 | AT1G80840 | Fw- CACCATGGATCAGTACTCATCCTC | Cloning into pEntr-D-TOPO |
|  |  | Rev- AAAAACTCTAACGGAATTTGAA |  |
| MYC2 | AT1G32640 | Fw- CACCATGACTGATTACCGGCTACA | Cloning into pEntr-D-TOPO |
|  |  | Rev- ACCGATTTTTGAAATCAAACTT |  |
| GUSpEntr | AT5G61250 | Fw- CACCTGTTACGTCCTGTAGAAACC | Cloning into pEntr-D-TOPO |
|  |  | Rev- TGCATCGGCGAACTGATCGT |  |
| RT-WRKY18 | AT4G31800 | Fw- TACTTGTAGCGACATACGAAG | qRT-PCR |
|  |  | Rev- CAGCAGCAAGAGCAGCTGTA |  |
| RT-WRKY40 | AT1G80840 | Fw- GTGGAGGATCAGTCCGTGTT | qRT-PCR |
|  |  | Rev- TGAAGCTGAACCACCATGAG |  |
| RT-At18S | AT3G41768 | Fw- AAACGGCTACCACATCCAAG | qRT-PCR |
|  |  | Rev- CCTCCAATGGATCCTCGTTA |  |
| RT-MYC2 | AT1G32640 | Fw- GCGTTGATGGATTTGGAGTT | qRT-PCR |
|  |  | Rev- TTGCTCTGAGCTGTTCTTGC |  |
| RT-AtDXS | AT4G15560 | Fw- CATATGATCAAGTTGTACATGAT | qRT-PCR |
|  |  | Rev- ACGTCACATCAAATGCTCCAC |  |
| RT-AtDXR | AT5G62790 | Fw- ATGGCCCGATAGAGTTCCTT | qRT-PCR |
|  |  | Rev- ACTGGCCTAGCACCAGAAGA |  |
| RT-AtCMS | AT2G02500 | Fw- CCAGCAATGGTGTGTCTCAA | qRT-PCR |
|  |  | Rev- ACACAGCATCGTATTCCCC |  |
| RT-AtCMK | AT2G26930 | Fw- CAGCAATGGTGTGTCTCAA | qRT-PCR |
|  |  | Rev- ACACAGCATCGTATTCCCCA |  |
| RT-AtMCS | AT1G63970 | Fw- GCGACGTTGATTCTCCAGAG | qRT-PCR |
|  |  | Rev- GCTTCTGTTTTCTCCGAGGC |  |
| RT-AtHDS | AT5G60600 | Fw- CGAGCAACCCAGTGATCATG | qRT-PCR |
|  |  | Rev- CCTGCCTCAGTAACTCCCAA |  |
| RT-AtGGPPS | AT1G49530 | Fw- TTCGAGCCGTTGGAGAATTG | qRT-PCR |
|  |  | Rev- GCCGCCGTTTTATGCAAATG |  |
| RT-SsDXS | JZ903931.1 | Fw- AGTGGAGGCCAATGGTTCTT | qRT-PCR |
|  |  | Rev- CCACCAATAAGTGACACAAC |  |
| RT-SsDXR | JZ903932.1 | Fw- GAAACACAGGAT TCATCCGTG | qRT-PCR |
|  |  | Rev- AGCTCGTCCAGCAGCATAAG |  |
| RT-SsHDS | JZ903936.1 | Fw- AGCACCGATAACCAAGTCGT | qRT-PCR |
|  |  | Rev- CAGCAGAGTTCATGCTGCAA |  |
| RT-SsGPPS | JZ903937.1 | Fw- GTGGTGGACATCAACTGCAC | qRT-PCR |
|  |  | Rev- AAAATGGCCCCCAAAACTAC |  |
| RT-SsCPS | JZ903938.1 | Fw- GCGAAGACCGATTTCAAGAG | qRT-PCR |
|  |  | Rev- CAGTCGCCAGGAAATAGGAA |  |
| RT-SsActina | JZ9223579.1 | Fw- GGTGCCCTGAGGTCCTGTT | qRT-PCR |
|  |  | Rev- GAGCCACCACTGAGGACAAT |  |
| 35S^2^-Fw | Gateway *vector* pGBW17 | GACTCTAGAGTTATCAACAAGT | PCR |
| WRKY18-Rev | AT4G31800 | TGTTGAAGCTATCAGTGACTG | PCR |
| WRKY40-Rev | AT1G80840 | GATGGATTGTCTCTAGTCACT | PCR |
| Myc2-Rev | AT1G32640 | GAGTTAAGCTCTCTCAACACT | PCR |

**Table S5**. List of primers used in this study
